# In vitro antibacterial effects of Tanreqing injection combined with vancomycin or linezolid against methicillin-resistant *Staphylococcus aureus*

**DOI:** 10.1186/s12906-018-2231-8

**Published:** 2018-05-30

**Authors:** Weifeng Yang, Jueling Liu, Biljana Blažeković, Yanan Sun, Shuhua Ma, Chuanyun Ren, Sanda Vladimir-Knežević, Chaohua Li, Yajun Xing, Guijie Tian, Yi Wang

**Affiliations:** 1 Experimental Research Center, China Academy of Chinese Medical Sciences, Nanxiao Road 16, Dongzhimen, Beijing, 100700 People’s Republic of China; 2 Department of Pharmacognosy, Faculty of Pharmacy and Biochemistry, University of Zagreb, Marulićev trg 20, 10000 Zagreb, Croatia; 3 ChuanYun Ren, Dongzhimen Hospital, Beijing University of Chinese Medicine, Haiyuncang alley 5, Dongcheng district, Beijing, 100700 People’s Republic of China; 4 Public health bureau of Tiexi district, Haifeng Road 2118, Tiexi district, Siping, 136000 People’s Republic of China

**Keywords:** Tanreqing, Vancomycin, Linezolid, Methicillin-resistant *Staphylococcus aureus*, MRSA, Synergistic effect, Biofilm

## Abstract

**Background:**

Combining conventional drugs and traditional medicine may represent a useful approach to combating antibiotic resistance, which has become a serious threat to global public health. This study aimed to evaluate the potential synergistic interactions between Tanreqing (TRQ) injection, a commercial traditional Chinese medicine formula used for the treatment of upper respiratory tract infection, and selected antibiotics used against methicillin-resistant *Staphylococcus aureus* (MRSA).

**Methods:**

The minimum inhibitory concentrations (MICs) of TRQ, vancomycin and linezolid against planktonic MRSA strain were determined by the broth microdilution method. The combined effects of TRQ and antibiotics were studied by the checkerboard method and the time-kill curve assay. The 2,3-bis-(2-methoxy-4-nitro-5-sulfophenyl)-2H-tetrazolium-5-carboxanilide (XTT) reduction assay was employed to determine the inhibitory effect of the test compounds alone and in combination against MRSA embedded in biofilms.

**Results:**

MRSA strain was found to be susceptible to TRQ formula with MIC value 4125 μg/ml, while the MIC values for antibiotics, vancomycin and linezolid, were 2.5 μg/ml. The checkerboard analysis revealed that TRQ markedly enhanced activities of the tested antibiotics by reducing their MICs. In the time-kill analysis, TRQ at 1/2 × MIC in combination with vancomycin at 1/2 × MIC, as well as TRQ at 1/8 × MIC in combination with linezolid at 1/2 × MIC decreased the viable colonies by ≥2log10 CFU/ml, resulting in a potent synergistic effect against planktonic MRSA. In contrast to the tested antibiotics, which did not affect mature MRSA biofilms at subinhibitory concentrations, TRQ alone showed strong ability to disrupt preformed biofilms and induce biofilm cell death. The combination of TRQ with vancomycin or linezolid at sub-MIC concentrations resulted in a synergistic antibiofilm effect significantly higher than for each single agent.

**Conclusions:**

This study provides the first in vitro evidence on the synergistic effects of TRQ and vancomycin or linezolid against planktonic and biofilm MRSA, and revealed their optimal combination doses, thereby providing a rational basis for the combination therapies against MRSA.

## Background

Antimicrobial resistance is recognized today as a major global public health concern that threatens the effective prevention and treatment of many infectious diseases representing an ever-increasing health and economic burden [1]. Methicillin-resistant *Staphylococcus aureus* (MRSA) is one of the most important pathogenic bacteria causing nosocomial and community acquired infections. MRSA infections constitute today a serious and still evolving public health challenge worldwide due to their high incidence and associated increased morbidity, the risk of mortality and medical costs [2–4]. As MRSA is resistant to methicillin and related β-lactams, the glycopeptide antibiotic vancomycin has long been the drug of choice for most MRSA infections. However, its widespread use resulted in reduced MRSA susceptibility as well as the emergence of a vancomycin-resistant strain of *S. aureus* [5, 6]. Several studies suggest that vancomycin may be less effective against serious MRSA infections with minimum inhibitory concentration (MIC) values at the higher end of the susceptibility and also associated the MIC increase with a substantial risk of vancomycin treatment failure and a higher mortality rate [7]. In addition, vancomycin-induced nephrotoxicity is an important adverse effect that can occur in patients following conventional and high doses of the drug [5, 8]. Linezolid is a newer antibacterial agent of the oxazolidinone class, which has a broad-spectrum of activity against the majority of common Gram-positive *cocci*. It has been found to be very useful for the treatment of MRSA infections where vancomycin has failed, but concerns about safety often limit its use. Also, the clinical reports have revealed the occurrence of linezolid resistant MRSA [6, 9]. Faced with the emergence of resistance to the last-resort antibiotics, their undesirable side effects, and general lack of new antibiotics, combination therapy has become an important new treatment strategy for MRSA infections. Additive and synergistic drug interactions can result in enhanced efficacy, delayed or reduced resistance, or minimized drug toxicity and side effects in patients.

Traditional Chinese medicine (TCM) has long been used in the Chinese population to treat various infectious diseases, and many prescriptions have been proved to be therapeutically effective [10, 11]. The Tanreqing (TRQ) injection is a Chinese herbal preparation made from Scutellariae radix (Huang Qin), Lonicerae flos (Jin Yin Hua), Forsythiae fructus (Lian Qiao), Ursi fel (Xiong Dan) and Naemorhedi cornu (Shan Yang Jiao). Recent phytochemical studies have revealed the characteristic chemical complexity of this TCM formulation. More than 50 different constituents belonging to the classes of flavonoids, phenolic acids, cholic acids and amino acids have been identified in the TRQ injection. Among these, five active ingredients (baicalin, ursodeoxycholic acid, chenodeoxycholic acid, chlorogenic acid, and caffeic acid) are selected as chemical markers for its quality control [12]. According to the TCM theory, TRQ formula clears heat, detoxifies and removes phlegm and it has been used in China to treat respiratory tract infections, pneumonia and chronic obstructive pulmonary disease [13]. In the clinical practice TRQ injection is usually administered intravenously, although it can also be given by nebulisation [14]. Due to the complexity of this multi-component mixture, its metabolism and the molecular mechanisms of activity are still poorly understood. No clinical pharmacokinetic data are available for the TRQ injection. The few published preclinical studies suggest rapid distribution of its major constituents from plasma to tissues and the organs, thus favoring the drug efficacy [15–17].

Clinical data demonstrated that TRQ is safe and well tolerated, and it effectively treats respiratory infections [18–20], by improving vancomycin efficacy in patients with MRSA pneumonia [21, 22]. However, in vitro studies on the antibacterial activity of this standardized formula are still scarce. Our recent study demonstrated that TRQ inhibits the growth of planktonic *Staphylococcus aureus* as well as bacterial cells embedded in a biofilm. [23]. Based on the previous findings, this study aimed to evaluate in vitro antibacterial activity of TRQ and its possible synergistic interactions with conventional antibiotics against MRSA in order to provide useful evidence for the rational and improved MRSA treatment.

## Methods

### Antimicrobial agents and chemicals

Tanreqing injection (TRQ, 33 mg/mL, lot no. Z20030054) was obtained from Shanhai Kaibao Pharmaceutical, China, vancomycin hydrochloride injection (lot no. 657) from Eli Lilly, Japan, and linezolid injection (lot no. 14K03U03) was from Pfizer, Norway. Tryptone and yeast extract were obtained from Oxoid, UK, NaCl from Merck, Germany, and 2,3,5-triphenyltetrazolium chloride (TTC) from Amresco, USA. 2,3-bis-(2-methoxy-4-nitro-5-sulfophenyl)-2H-tetrazolium-5-carboxanilide (XTT) and phenazine methosulfate (PMS) were purchased from Sigma-Aldrich, USA.

### Bacterial strain and growth conditions

Methicillin-resistant *Staphylococcus aureus* (MRSA, ATCC 43300) was purchased from the American Type Culture Collection (ATCC, USA) and maintained in cryogenic storage at − 80 °C on glass beads. Working culture of bacteria was maintained on an agar slant at 4 °C and subcultured in Luria-Bertani (LB) broth (10 g tryptone, 5 g yeast extract, 5 g NaCl per liter) prior to use. The bacterial culture maintained on agar slant at 4 °C was subcultured in Luria-Bertani (LB) broth (10 g tryptone, 5 g yeast extract, 5 g NaCl per liter) prior to use. Briefly, a single MRSA colony was inoculated into the LB broth and incubated for 24 h at 37 °C with shaking (280 rpm). An overnight broth culture was diluted with fresh medium to obtain a starting inoculum of about 5 × 10^6^ CFU/ml.

### Determination of the minimal inhibitory concentration

The minimal inhibitory concentrations (MICs) of TRQ and antibiotics were determined by a standard broth microdilution method in sterile 96-well microplates [24]. Two-fold serial dilutions were made in LB broth over a range to give final concentrations of 16500–33 μg/ml for TRQ, and 20–0.039 μg/ml for vancomycin and linezolid, respectively. One hundred microliters of bacterial suspension (5 × 10^6^ CFU/ml) was added to each well. Negative control comprised LB broth and tested sample while the positive control was LB broth and bacterial suspension only. The final volume of each well was 200 μl. The MICs of test samples were detected after 24 h incubation at 37 °C, following addition of 20 μl of 2.5 mg/ml TTC and incubation for an additional 20 min at 37 °C. Viable bacteria reduced the yellow dye to pink. MIC was defined as the lowest sample concentration that prevented this change and exhibited complete inhibition of microbial growth [25].

### Checkerboard assay

The combined effect of TRQ with the vancomycin or linezolid was evaluated by checkerboard microdilution method from which the fractional inhibitory concentration index (FICI), the predictor of the type of interaction between compounds, was obtained [26]. The TRQ was diluted two-fold in vertical orientation, while the antibiotic was diluted two-fold in horizontal orientation in the 96-well microtiter plates using LB broth. The concentrations tested for every combination of TRQ-antibiotic were their respective MICs, followed by 1/2, 1/4, 1/8, and 1/16 of the MICs values, respectively. The final volume of each well was 100 μl comprising 50 μl of each tested sample dilution. Subsequently, 100 μl of the bacterial suspension (5 × 10^6^ CFU/ml) was added to all wells. Negative controls were LB broth and the TRQ/antibiotic combination while positive controls were media and bacterial suspension. After 24 h incubation at 37 °C, 20 μl of TTC (2.5 mg/ml) was added to each well and the plates were incubated again for 20 min. Wells containing the solution which turned pink comparable to that of the positive control were interpreted as positive for bacterial growth, while the wells containing colorless solutions were interpreted as negative for bacterial growth. The FICI for the combination with negative results were calculated as follows: FICI = FIC A + FIC B, where FIC A is the MIC of drug A in the combination/MIC of drug A alone, and FIC B is the MIC of drug B in the combination/MIC of drug B alone. The results were interpreted as synergy (FICI ≤0.5), addition (0.5 < FICI ≤1), indifference (1 < FICI ≤2) or antagonism (FICI > 2) [27, 28]. The experiments were performed in triplicate and the median FICI values were used in the analysis.

### Time-kill curve assay

The time-kill analysis was used to determine the growth of MRSA in the presence of TRQ, vancomycin and linezolid, used as monotherapies or in combinations, for 24 h. Based on MIC and FICI value for each drug, the concentrations of vancomycin and linezolid were set to 1/2 × MIC, while TRQ was assessed at 1/2 × MIC and 1/8 × MIC, respectively. Then, bacterial solution was added to each solution (5 × 10^6^ CFU/ml, 6 ml), and incubated at 37 °C with shaking. The growth control flasks contained only bacteria and 6 ml LB broth. At predetermined time points (0, 2, 4, 6, 8, 10, 12 and 24 h), 10 μl of the samples were taken aseptically for bacterial counts. Each aliquot was serially diluted, plated onto LB agar plates and incubated for 24 h at 37 °C in order to determine the number of CFU/ml. The colonies were counted only on plates that had between 30 and 300 colonies. Subsequently, time-kill curves were generated by plotting the log10 CFU/ml against the time (h). Concentrations of each tested sample with negative 24 h-antibacterial effects were selected for synergistic studies. The time-kill assays on the TRQ/antibiotic combinations were performed in the same manner as described above. Time-kill curves were then constructed as a function of time and the results were interpreted by the effect of the combination in comparison with the most active single drug alone. The interaction was defined as the log10 CFU/ml increase in killing at 24 h (ΔLC24) as follows: ΔLC24 ≥ 2 log10 CFU/ml, synergy; ΔLC24 = 1–2 log10 CFU/ml, additivity; ΔLC24 = ±1 log10 CFU/ml, indifference; and ΔLC24 > − 1 log10 CFU/ml, antagonism [29]. Bactericidal activity is defined as a ≥ 3-log reduction in the initial CFU count within 24 h.

### Biofilm assay

The preparation of a mature biofilm of MRSA was performed according to our previous work [23]. Briefly, the overnight culture of MRSA in LB medium supplemented with 0.25% glucose (LB-G) was diluted in the respective medium to an optical density at 600 nm (OD600) of 0.05. Then, 200 μl aliquots of bacterial solutions were placed into a 96-well microtiter plate, while the wells containing medium served as negative controls. After 24 h of incubation at 37 °C, media and planktonic cells were removed and wells containing biofilms were rinsed with 200 μl of 0.9% NaCl. Antibiotic agents diluted in LB-G medium at different concentrations, based on the result of checkerboard method, were tested as single drugs and in combinations by adding 200 μl of the tested sample to each well. Wells containing medium only served as negative controls. Following 24 h incubation at 37 °C, the wells were washed to remove planktonic cells and ensure that only cells within biofilms were assayed. Then the XTT reduction assay was performed to evaluate the viability of the biofilms. For this purpose, 40 μl of the XTT-PMS solution (200 mg/ml XTT, 2 μM PMS) was added to each well, plates were incubated for 2 h at 37 °C in the dark and the optical density was then measured at 490 nm using a microplate reader. A decrease in the number of live cells correlates with a decrease in overall activity of the dehydrogenases responsible for transforming the sodium salt of tetrazolium XTT into formazan which was determined colorimetrically. All experiments were repeated three times.

### Statistical analysis

All the experiments were performed in triplicate. Data were expressed as mean ± standard deviation. Statistical analyses and significance, as measured by the Student’s *t*-test for paired samples were performed using Prism software version 4.0 (GraphPad Software, CA, USA). In all comparisons, *p* < 0.05 was considered statistically significant.

## Results

### Antibacterial activities of TRQ and combined effect with vancomycin and linezolid on MRSA

The antibacterial activity of TRQ, vancomycin and linezolid against MRSA was assessed using the broth microdilution method and the results are shown in Table 1. The MRSA strain was susceptible to TRQ formula with MIC value 4125 μg/ml, while the MIC values for both tested antibiotics were 2.5 μg/ml.

**Table 1:** Minimal inhibitory concentration (MIC) and fractional inhibitory concentration index (FICI) of Tanreqing, vancomycin and linezolid against MRSA

Strain	Agent	Minimal inhibitory concentration (μg/ml)		FICI	Outcome
		Alone	Combination		
MRSA ATCC 43300	Tanreqing Vancomycin	4125 2.5	2063 1.25	1	Addition
	Tanreqing Linezolid	4125 2.5	516 1.25	0.625	Addition

Results are means of three different experiments. The results were interpreted as synergy (FICI ≤0.5), addition (0.5 < FICI ≤1), indifference (1 < FICI ≤2) or antagonism (FICI > 2)

Table 1 also summarizes the results of microbial growth inhibition by the combination of TRQ and vancomycin and TRQ and linezolid, respectively, determined by the checkerboard method. As evident from the table, TRQ markedly enhanced the activities of the tested antibiotics by reducing their MICs. The MIC value for vancomycin was reduced from 2.5 μg/ml to 1.25 μg/ml when used in combination with TRQ. Likewise, a 2-fold MIC reduction was found for linezolid in the presence of TRQ. Reduction in MIC of TRQ in combination with antibiotics was also observed. When combined with vancomycin, the MIC of TRQ was reduced 2-fold, while the combination with linezolid resulted in an 8-fold reduction of its MIC. According to the calculated FICI values (Table 1), the interactions of TRQ and vancomycin as well as of TRQ and linezolid were all classified as additive (0.5 < FICI ≤1.0).

### Time-kill curve analysis of TRQ, vancomycin and linezolid and their combinations against MRSA

**Fig. 1:**
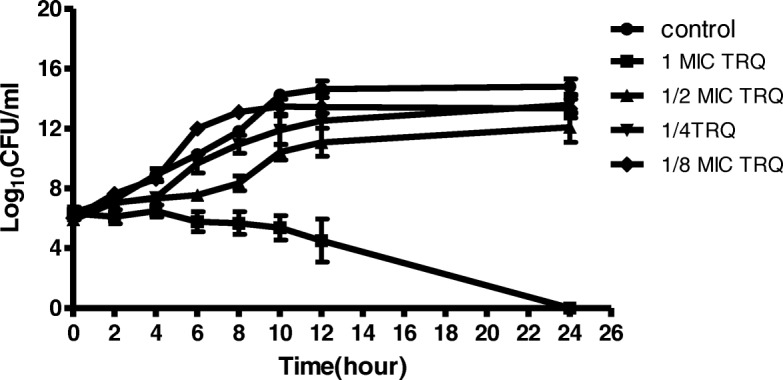
Activity of TRQ at different concentrations in a time-kill analysis against MRSA

An evaluation of the dynamic relationship between TRQ and MRSA was performed by time-kill experiments and the results are shown in Fig. 1. At concentration 1 × MIC, TRQ had strong growth inhibitory effects on MRSA. A large drop in colony counts observed during 12 h (> 8 log CFU/ml) resulted in bacterial eradication within 24 h. However, the ability of TRQ to inhibit microbial growth decreased with reducing its concentration. At concentration 1/2 × MIC, TRQ showed inhibitory effect on the bacteria but was followed by bacterial regrowth after 6 to 24 h of incubation. TRQ at concentrations 1/4 × MIC and1/8 × MIC showed a similar tendency as the control group.

**Fig. 2:**
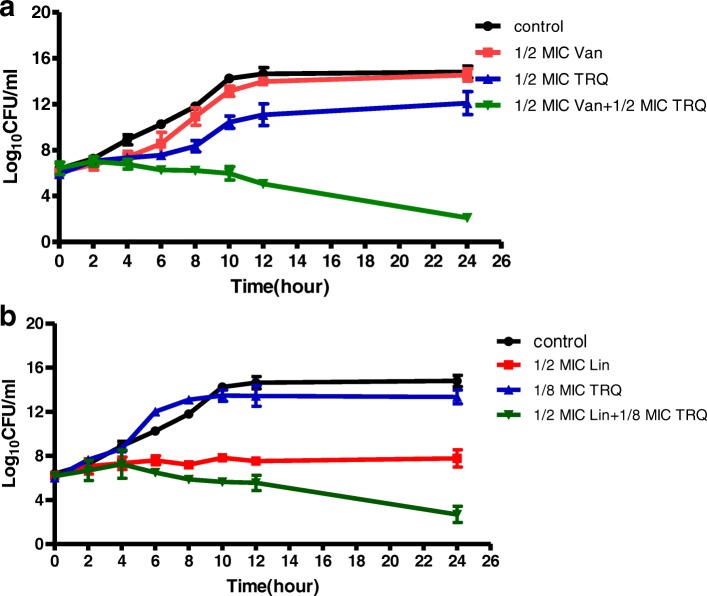
Time-kill curves of TRQ, vancomycin and linezolid alone and in combinations against MRSA (A. TRQ with vancomycin; B. TRQ with linezolid)

The findings of time-kill studies for the planktonic MRSA strain during exposure to combination of TRQ and antibiotics are presented in Fig. 2. Figure 2a shows the effect of TRQ at concentration 1/2 × MIC and vancomycin at concentration 1/2 × MIC, used alone and in combination against MRSA. At the subinhibitory concentrations tested, the two antibacterial agents used alone showed little (TRQ) or no activity (vancomycin) on bacterial growth. However, combination of 1/2 × MIC vancomycin with 1/2 × MIC TRQ induced a strong antibacterial effect leading to eradication of MRSA during 24 h. The combination of TRQ/ vancomycin increased the killing effect by 9.32 (ΔLC24 > 2 log_10_ CFU/ml) at 24 h, compared with the most active single drug TRQ, which demonstrated that the TRQ had a synergistic effect with vancomycin against MRSA. The time-kill curves of TRQ and linezolid alone and in combination are shown in Fig. 2b. Linezolid alone at concentration 1/2 × MIC showed antibacterial effect from 0 h to 12 h. However, after that the growth rate increased rapidly. In contrast, combination of TRQ at its 1/8 × MIC and linezolid (1/2 × MIC) greatly reduced the viable counts of bacteria during 24 h when compared with the most active agent used alone. The combination of TRQ with linezolid resulted in a potent synergistic effect on MRSA with ΔLC24 = 6.38. Taken together, TRQ combined with conventional antibiotics at subinhibitory concentrations resulted in strong, long-lasting synergistic antibacterial effects. In addition, the two combinations reduced the viable count by > 3 log_10_ CFU/ml when compared with the starting inoculum, demonstrating a bactericidal effect in this way.

### Effect of TRQ alone and in combination with vancomycin and linezolid on MRSA biofilm

**Fig. 3:**
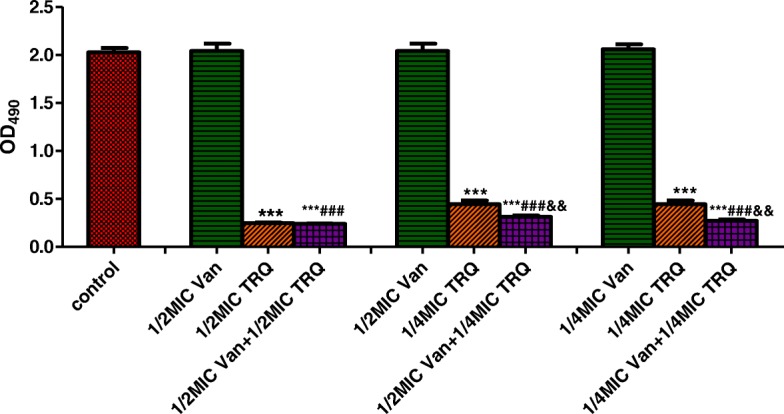
Effect of TRQ combined with vancomycin on the preformed MRSA biofilm. Results are presented as the mean ± standard deviation of triplicate assays. (***p < 0.001 vs. control group; ###*p* < 0.001 vs. vancomycin group; &&*p* < 0. 01 vs. TRQ group)

**Fig. 4:**
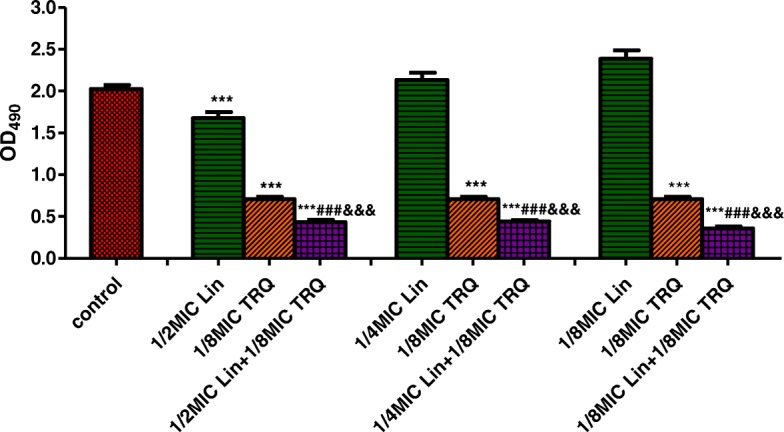
Effect of TRQ combined with linezolid on the preformed MRSA biofilm. Results are presented as the mean ± standard deviation of triplicate assays. (***p < 0.001 vs. control group; ###p < 0.001 vs. linezolid group; &&&p < 0. 001 vs. TRQ group)

The antibiofilm effect of TRQ alone and in combination with antibiotics was studied against MRSA embedded in the mature biofilm by evaluating the metabolic activity of the biofilm cells and the results are presented in Figs. 3 and 4. As shown in Fig. 3, treatment with vancomycin alone at sub-MIC concentrations did not disrupt preformed biofilms and did not significantly induce biofilm cell death. In contrast, TRQ alone at concentrations 1/2 × MIC or 1/4 × MIC was able to cause a strong statistically significant reduction of the viability of bacteria in mature biofilms, compared to the untreated control biofilm. The combination of 1/2 × MIC TRQ with 1/2 × MIC or 1/4 × MIC vancomycin resulted in a synergistic antibiofilm effect significantly higher than for each single agent (*p* < 0.001 and *p* < 0.01, respectively).

Figure 4 displays the results of antibiofilm activity of TRQ alone and in combination with linezolid. When used at concentration 1/2 × MIC, linezolid reduced the viability of mature MRSA biofilm*,* while at 1/4× and 1/8 × MIC this antibiotic did not significantly affect the growth of biofilm, compared with untreated control biofilm. In contrast, TRQ at tested concentration 1/8 × MIC showed strong activity against cells of the MRSA biofilm. A synergistic effect has been observed when subinhibitory concentrations of both TRQ (1/8 × MIC) and linezolid (1/2–1/8 × MIC) were combined together to treat mature MRSA biofilms (*p* < 0.001).

## Discussion

Inappropriate antibiotics therapy and overuse of antibiotics together with the lack of novel, more effective drugs has greatly contributed to the emergence of multidrug-resistant bacteria. Chinese herbal drugs with a long tradition of use in therapy of infectious diseases may serve as sources of new antimicrobial agents or, in combination with conventional antibiotics, may complement conventional therapies and so offer a promising strategy to overcome bacterial resistance mechanisms and restore the effectiveness of antibiotics.

To our knowledge, this investigation for the first time reports the in vitro antimicrobial effect of the TRQ traditional formula against multidrug-resistant bacteria. MRSA was found to be sensitive to TRQ, although to a much lesser extent than to commonly used anti-MRSA antibiotics, vancomycine and linezolid. Our study showed that, in addition to its weak antibacterial effect against MRSA, TRQ also possess the potential to enhance the activity of vancomycine and linezolid against that serious pathogen. The effect of TRQ injection combined with antibiotics on planktonic MRSA was remarkably higher than that of single antibiotics.

The accurate prediction of synergy between a commercial antibiotic and a natural product, based upon the results of in vitro testing, is crucial for the determination of optimal doses of agents used in the combination antimicrobial therapy [30]. In this study we applied two most widely used methods, the checkerboard method and time-kill curve assay, to determine the interactions between TRQ and vancomycin or linezolid. The results of the checkerboard tests showed decrease in the MIC when combining TRQ with each of the tested antibiotics, which indicates the ability of TRQ to enhance the antibacterial activity of those antibiotics against MRSA due to additive interaction. The time-kill curves of vancomycin and linezolid revealed that both drugs were non-concentration dependent antibiotics, which coincides with previous reports [31, 32]. Most importantly, the resulting time-kill data revealed that TRQ can greatly increase the susceptibility of MRSA strain toward vancomycin and linezolid. At subinhibitory concentrations, TRQ was found to be able to synergistically increase the antibacterial activity of the tested antibiotics, ensuring their prolonged effectiveness. As evident, the time-kill studies demonstrated a higher degree of synergy, as did the checkerboard analysis. However, the results obtained in the comparative checkerboard and time-kill studies have often been at odds because each method measures different parameters [33]. Although the checkerboard method is simple to perform, it is based upon MICs which reflect the inhibition of bacterial growth only. Since the results are usually examined only at one point in time, this method typically provides a static view of antimicrobial interaction, whereas the FIC indices calculation and interpretation incorrectly assume that all antimicrobials have linear dosage-response curves. On the other hand, the time-kill curve assay measures the microbicidal activity of a drug combination and, based on serial colony counts, provides a dynamic picture of antimicrobial action and interaction over time, giving more detailed data, including the rate and extent of antibacterial activity of drug combinations [34]. Although the experiments are labor-intensive and time-consuming, the time-kill method is considered as a more reliable predictor of in vivo synergy [33].

The ability of MRSA to form biofilms greatly contributes to its survival and virulence, thus limiting the possible therapeutic options. It is well-known that bacteria grown in biofilms differ greatly from the same organisms grown in suspensions by having different growth characteristics and taking up nutrients and drugs differently [35]. Therefore, besides activity against planktonic bacteria, in this study we have also evaluated the efficacy of combined TRQ/antibiotic therapy against MRSA embedded in biofilm. To quantify biofilm growth, metabolic activity within the biofilm was measured using XTT reduction assays, which are known to correlate with biofilm CFU [36]. The obtained results proved that subinhibitory concentrations of TRQ are very effective against mature MRSA biofilms, supporting the previous report on strong antibiofilm properties of TRQ [23]. Most importantly, we have found that TRQ can greatly increase antimicrobial susceptibility of bacteria grown in biofilms to conventional antibiotics. While vancomycin or linezolid alone at subinhibitory concentrations did not significantly affect the growth of biofilms, their combination with this traditional Chinese drug which potently enhanced their antibiotic action against biofilms showed a great promise in dispersing existing MRSA biofilms.

TRQ injection is a multicomponent Chinese herbal drug rich in five active compounds, baicalin, ursodeoxycholic acid, chenodeoxycholic acid, chlorogenic acid and caffeic acid, which are considered important for its quality evaluation [12]. Interestingly, all of them are reported to possess a wide range of bioactivity, including antibacterial properties. As major constituents of Scutellariae baicalensis radix, a well-known TCM herbal drug used in the treatment of inflammation, cardiovascular diseases, bacterial and viral infections, baicalin and its aglycone baicalein have been studied extensively [37]. Baicalin or baicalein alone showed moderate antibacterial activity against resistant *S. aureus* strains with MIC values from 64 to 256 μg/ml [38–40]. Moreover, when used at concentrations lower than their MICs in combination with b-lactams, tetracycline, oxytetracycline and ciprofloxacin, both flavones showed remarkable synergies and greatly increased the susceptibility of MRSA to those antibiotics, demonstrating the ability to restore their effectiveness. The synergistic effect of baicalin/baicalein on MRSA may involve multiple mechanisms of action, such as inhibition of efflux pumps like NorA, which are able to efflux a range of antibiotics, or by interfering with the cell wall integrity through direct binding to peptidoglycan structure [39, 41–43]. Baicalein may also act as an inhibitor of bacterial resistant related enzymes such as penicillinase, associated with penicillin resistance in penicillinase-producing MRSA [44]. Furthermore, baicalein was shown to inhibit the MRSA pyruvate kinase, an enzyme essential for *S. aureus* growth and survival, as well as to reduce the activity of virulence factors produced by *S. aureus*, such as α-hemolysin which might contribute to its antibacterial actions [45]. Recently, Chen et al. reported that baicalein possesses anti-biofilm properties, demonstrating its ability to inhibit *S. aureus* biofilm formation, disrupt the already formed biofilms and enhance vancomycin permeability, reduce the production of staphylococcal enterotoxin A and α-hemolysin, and inhibit the quorum sensing system [46]. Chlorogenic and caffeic acids are phenolic acids which were found to be effective against both Gram-negative and Gram-positive bacteria, including *S. aureus*, showing antistaphylococcal properties on both planktonic and biofilm cells. They have been shown to exert their antibacterial action by disrupting membrane function through increased cell membrane permeability, depolarized cell membranes, and reduced respiratory activity, which can lead to cell death [47, 48]. It is important to highlight that subinhibitory concentrations of those phenolic acids attenuate virulence and hamper *S. aureus* infections through inhibition of different secreted and/or cell wall-related virulence factors [48–50]. Lima et al. [51] reported that caffeic acid potentiated the antibiotic activity of norfloxacin, imipenem and gentamicin against *Staphylococcus aureus, Escherichia coli* and *Pseudomonas aeruginosa* strains. Besides polyphenolic constituents of TRQ, bile acids originating from animal drugs are also reported to possess antibacterial activity [52, 53]. However, it is also worth to mention that some pharmacologically active compounds used today in the treatment of non-infectious conditions are having antimicrobial activities and are acting in different manners on microbial growth. Known as non-antibiotics, several anti-inflammatory, neurotropic, diuretic, antispasmodic and mucolytic agents might themselves be effective and/or potentiate the efficacy of certain conventional antibiotics [54–56]. Therefore, it has to be bear in mind the co-administration of antibiotics and plant-derived non-antibiotics could also contribute to reducing resistance and enhancing drug activity.

The use of traditional Chinese herbal drugs in the treatment of infectious diseases may have some advantage due to their quite different mechanism of activity from the antibiotics. Chinese herbal formulas used for treating infections usually exhibit anti-inflammatory activity in addition to their antimicrobial effects. Herbal drugs classified in TCM as heat-clearing and detoxifying, such as Scutellariae radix, Coptidis rhizoma, Lonicerae flos, Forsythiae fructus etc., are common constituents of the preparations of this type. Modern pharmacological studies revealed that they can exert anti-inflammatory effect through different mechanisms of action, including inhibition of inflammatory cytokines and mediators, blocking of inflammatory signaling, and interfering with chemokines. At the same time, they are acting as antimicrobials through inhibition of microbial adherence to mucosal or epithelial surfaces, inhibition of endotoxin shock, and selective inhibition of microbial growth [57]. Additionally, the antipyretic properties of TRQ formula have been also reported [58]. Therefore, a novel approach to the treatment of MRSA infections based on the combination of existing antibiotics with traditional herbal drugs displaying multiple mechanisms of action, such as TRQ, may provide enhanced clinical benefits and be helpful in tackling antimicrobial resistance.

## Conclusions

In summary, our findings revealed that TRQ in combination with vancomycin or linezolid has synergistic antibacterial effects against MRSA in planktonic form and biofilms, making the antibiotics more effective and extending their antibacterial action. Due to its ability to potentiate the effects of current antibiotics, this study supports TRQ as an effective treatment strategy for MRSA-associated infections.

## Abbreviations

ATCC: American type culture collection
FICI: Fractional inhibitory concentration index
LB: Luria-Bertani
MICs: Minimum inhibitory concentrations
MRSA: Methicillin-resistant *Staphylococcus aureus*
PMS: Phenazine methosulfate
TCM: Traditional Chinese medicine
TRQ: Tanreqing injection
TTC: 2,3,5-triphenyltetrazolium chloride
XTT: 2,3-bis-(2-methoxy-4-nitro-5-sulfophenyl)-2 h-tetrazolium-5-carboxanilide
